# Supplementary material for: Identification and functional analysis of non-coding regulatory small RNA FenSr3 in Bacillus amyloliquefaciens LPB-18
Source: PeerJ. 2023 May 15;11:e15236. doi: 10.7717/peerj.15236 (PMC10194069; doi:10.7717/peerj.15236)
Supplement: Supplemental Information 4 [file peerj-11-15236-s004.zip › KO/CK-vs-T1_map/map01521.html]

KEGG PATHWAY: EGFR tyrosine kinase inhibitor resistance - Reference pathway


|  |  |
| --- | --- |
| **EGFR tyrosine kinase inhibitor resistance - Reference pathway** |  |

[
Pathway menu
| Organism menu
| Pathway entry
| Show description
| User data mapping
]

EGFR is a tyrosine kinase that participates in the regulation of cellular homeostasis. EGFR also serves as a stimulus for cancer growth. EGFR gene mutations and protein overexpression, both of which activate down- stream pathways, are associated with cancers, especially lung cancer. Several tyrosine kinase inhibitor (TKI) therapies against EGFR are currently administered and are initially effective in cancer patients who have EGFR mutations or aberrant activation of EGFR. However, the development of TKI resistance is common and results in the recurrence of tumors. Studies over the last decade have identified mechanisms that drive resistance to EGFR TKI treatment. Most outstanding mechanisms are: the secondary EGFR mutation (T790M), activation of alternative pathways (c-Met, HGF, AXL), aberrance of the downstream pathways (K-RAS mutations, loss of PTEN), impairment of the EGFR-TKIs-mediated apoptosis pathway (BCL2-like 11/BIM deletion polymorphism), histologic transformation, etc.

|  |  |  |
| --- | --- | --- |
| Reference pathway | 184% 150% 122% 100% 82% 67% 55% | 图片下载 |
